# The relationship between oxidant levels and gut physiology in a litter-feeding termite

**DOI:** 10.1038/s41598-018-37043-2

**Published:** 2019-01-24

**Authors:** Gessica Sousa, Ana Caroline P. Gandara, Pedro L. Oliveira, Fabio M. Gomes, Ana Cristina Bahia, Ednildo A. Machado

**Affiliations:** 1 Laboratório de Bioquímica de Insetos e Parasitos (Labip), Instituto de Biofísica Carlos Chagas Filho, Universidade Federal do Rio de Janeiro, Rio de Janeiro, Brazil; 2 Laboratório de Bioquímica de Artrópodes Hematófagos, Instituto de Bioquímica Médica Leopoldo de Meis, Universidade Federal do Rio de Janeiro, Rio de Janeiro, Brazil; 3 Laboratory of Malaria and Vector Research, National Institute of Health, Bethesda, United States of America; 4 Instituto Nacional de Ciência e Tecnologia em Entomologia Molecular (INCT-EM), Rio de Janeiro, Brazil

## Abstract

The termite gut is an efficient decomposer of polyphenol-rich diets, such as lignocellulosic biomasses, and it has been proposed that non-enzymatic oxidative mechanisms could be involved with the digestive process in these animals. However, oxidant levels are completely unknown in termites, as well as protective mechanisms against oxidative damage to the termite gut and its microbiota. As the first step in investigating the role oxidants plays in termite gut physiology, this work presents oxidant levels, antioxidant enzymatic defenses, cell renewal and microbiota abundance along the litter-feeding termite *Cornitermes cumulans* gut compartments (foregut, midgut, mixed segment and hindgut p1, p3, p4, and p5 segments) and salivary glands. The results show variable levels of oxidants along the *C*. *cumulans* gut, the production of antioxidant enzymes, gut cell renewal as potential defenses against oxidative injuries and the profile of microbiota distribution (being predominantly inverse to oxidant levels). In this fashion, the oxidative challenges imposed by polyphenol-rich diet seem to be circumvented by the *C*. *cumulans* gut, ensuring efficiency of the digestive process together with preservation of tissue homoeostasis and microbiota growth. These results present new insights into the physicochemical properties of the gut in a litter-feeding termite, expanding our view in relation to termites’ digestive physiology.

## Introduction

Lignocellulosic biomass is the most abundant and widespread renewable energy resource on Earth, being mainly composed of cellulose, hemicellulose and lignin polymers^1,2^. Hence, it is the most promising source for second-generation biofuels production^3^. However, due to the prevalence of highly recalcitrant C-C bonds, lignocellulose polysaccharides are protected from industrial enzymatic degradation^1^. Similarly, most organisms are unable to digest lignocellulose. Termites (Insecta: Blattodea), however, are bioreactor models for biomass and lignocellulose deconstruction^4^, digesting 65–87% of hemicellulose and 74–99% of cellulose present in the ingested food^5^. Beyond lignin, a great diversity of other polyphenols, such as tannins, can also be found in the termite diet, especially in litter-feeders.

Effective lignocellulose degradation requires combined enzymatic attack of hydrolases, esterases, peroxidases and oxidases^6–11^, as well as non-enzymatic oxidative mechanisms (known in fungi), which are mostly mediated by reactive oxygen species (ROS)^12,13^. ROS are radical and non-radical derivatives of molecular oxygen^14^, which can oxidize polyphenols, such as lignin and tannins, and reduce cellulose crystallinity^15,16^. As there are only few published reports identifying oxidases in termites^7,9^, it has been proposed that non-enzymatic oxidant molecules might be critical to the gut physiology in these animals^17,18^. The termite polyphenol-rich diet can induce the production of significant quantities of oxidants in the gut lumen, as previously observed in other insect models^15,19,20^. The production of oxidant molecules (e.g., ROS) is also a key factor in the regulation of insect immunity, as they have been shown to control the intestinal microbiota growth and pathogens ingested during the feeding^21–23^.

Oxidants generated in the gut lumen might diffuse through the epithelial layer representing a dangerous oxidative risk to the cells. Hence, antioxidant enzymes appear to be essential in protecting the termite gut, as previously shown in another insect model^19^. Superoxide dismutase (SOD) and glutathione peroxidase (GPX) are part of the first line of antioxidant defense in animal cells. SOD catalyses dismutation of the highly reactive superoxide anion (O_2_^•−^) to the less reactive hydrogen peroxide (H_2_O_2_), which is then converted to water by GPX^24^. Besides antioxidant defenses, the termite gut secretes a peritrophic membrane around the food bolus that acts as a physical barrier against abrasive and chemical (including oxidants) components^25,26^. Apart from these defenses, gut epithelial cells require substantial regenerative capabilities in order to withstand the stress generated by oxidant molecules^27,28^.

The digestive system of Termitidae (termites with no protists in the gut) is composed of a pair of salivary glands and four gut compartments: foregut, midgut, mixed segment and hindgut. The hindgut is further differentiated into ileum (p1), enteric valve (p2), paunch (p3), colon (p4) and rectum (p5)^29^. Because of their metabolic roles, the distribution of microbiota throughout these gut compartments is dependent on several physiological factors, such as gut luminal pH, O_2_ and H_2_ levels and redox potential^17,30–34^. Consequently, the high digestive efficiency of termites is the result of successful association between host and microbiota enzymes at different intestinal segments^9,35^.

*Cornitermes cumulans* (Kollar, 1832) (Blattodea: Termitidae) is a litter-feeding, soil-mound building termite. It digests lignocellulose from litter and ingests soil for nest building^36^. Apart from the structural organization of the digestive system^37^ and gut microbiota composition^38,39^, there are no other descriptive reports of the gut physiology in *C*. *cumulans*. Oxidant levels and the protective mechanisms against them are therefore completely unknown in this litter-feeder, as well as in other termite species. We hypothesized that the levels of oxidant molecules in the *C*. *cumulans* gut impact the microbiota distribution, as well as antioxidant enzyme activities and cell turnover along the digestive system of this insect. Our results indicate that oxidant levels vary across gut compartments in a manner predominantly contrary to microbiota abundance profiles. Additionally, this variance coupled with localized increases in epithelial regeneration may be a strategy to balance efficiency of digestion while ensuring tissue homeostasis. Such oxidants might be involved in the digestion of polyphenols or, alternatively, might be generated from the digestive process.

## Results

### General organization of *C*. *cumulans* digestive system

**Figure 1:**
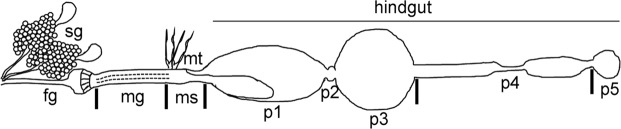
General organization of *C*. *cumulans* digestive system by line drawing. The alimentary canal is differentiated into foregut (fg), midgut (mg) containing the peritrophic membrane (dotted line), mixed segment (ms) and five hindgut compartments: ileum (p1), enteric valve (p2), paunch (p3), colon (p4) and rectum (p5). A pair of salivary glands (sg) is attached to the foregut and Malpighian tubules (mt) mark the transition between midgut and mixed segment.

Similar to findings in other Termitidae^29^, the *C*. *cumulans* digestive system consists of an alimentary canal differentiated into foregut, midgut, mixed segment and five hindgut compartments (p1, p2, p3, p4 and p5), and a pair of salivary glands (Fig. 1). In this work, due to technical reasons, the mixed segment and hindgut p1 compartment were considered a single sample and p2 segment (a valve with no expected role in the synthesis and secretion of digestive enzymes) was disregarded. To better understand the gut organization, size and dry weight of each digestive segment were measured. While workers had average body lengths of 5.2 ± 0.26 mm, their guts were much longer, measuring 16.09 ± 0.45 mm (Table 1). P4 was the longest gut compartment (5.56 ± 0.13 mm) and p2 the shortest (0.17 ± 0.01 mm). For dry weight, digestive segments were evaluated with and without the food bolus, which was the heaviest component of each compartment (Table 1). Considering the ratio between weight and size, p3 was the heaviest gut compartment (7.19 mg/mm) and p4 the lightest (0.62 mg/mm).

**Table 1:** Size and dry weight measures of *C*. *cumulans* digestive segments.

Digestive segment	Size (mm)	Dry weight with food (mg)	Dry weight without food (mg)	Difference (mg)	Ratio (mg/mm)
sg	nd	nd	1.25 ± 0.31	nd	nd
fg	2.7 ± 0.12	2.33 ± 0.33	1.02 ± 0.30	1.31	0.86
mg	2.12 ± 0.08	3.52 ± 0.63	1.95 ± 0.16	1.57	1.66
p1	3.61 ± 0.09	13.88 ± 1.83	2.45 ± 0.45	11.43	3.84
p2	0.17 ± 0.01	nd	nd	nd	nd
p3	2.01 ± 0.04	14.47 ± 1.83	2.02 ± 0.25	12.45	7.19
p4	5.56 ± 0.13	3.47 ± 0.42	3.2 ± 0.34	0.27	0.62
p5	1 ± 0.03	1.67 ± 0.17	1.2 ± 0.40	0.47	1.67
total gut	16.09 ± 0.45				
termite	5.2 ± 0.26				

Size values are mean ± S.E.M. from 10 worker guts. Dry weight values are from six samples of three different termite mounds (two replicates each and a pool of 30 termites per replicate). “Difference” column comprehends the values of “dry weight with food” minus “dry weight without food”; “ratio” column consists in the values of “dry weight with food” divided by “size”. Salivary glands (sg), foregut (fg), midgut (mg), hindgut compartments – p1, p2, p3, p4 and p5, not determined (nd).

### Oxidant levels along the alimentary canal

**Figure 2:**
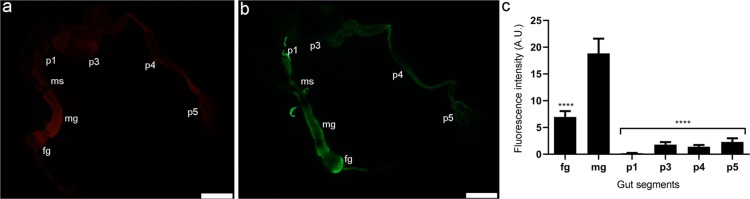
Profile of oxidant levels throughout *C*. *cumulans* alimentary canal. (**a**) Representative gut labelled with DHE or (**b**) CM-H_2_DCFDA and analysed under a fluorescence stereomicroscope. Foregut (fg), midgut (mg), mixed segment (ms) and hindgut compartments – p1, p3, p4 and p5. Bars = 1 mm. (**c**) Fluorescence intensity in arbitrary units (A. U.) of densitometry analysis of DHE labelled guts; ****p = < 0.0001. Fg *vs*. p1 compartment and fg *vs*. p4 were also significantly different, p = 0.0045 and 0.0314, respectively. The other combinations presented no significance. The data shown are mean ± S.E.M. from ten replicates with ANOVA and Tukey’s multiple comparisons test.

The oxidant levels throughout the *C*. *cumulans* alimentary canal were monitored by stereomicroscopy using the oxidant-sensitive fluorescence probes DHE (Fig. 2a – additional images are provided in Fig. S1) or CM-H_2_DCFDA (Fig. 2b). The strongest fluorescence signal was detected in the midgut, but it was also high in the foregut. The signal was reduced in the mixed segment, almost absent in hindgut p1 and p3 segments and present at lower levels in p4 and p5 compartments (Figs 2 and S1). In addition, guts were analysed at finer details under confocal microscopy (Fig. S2) and autofluorescence was checked and considered for quantification analysis.

### Antioxidant enzymatic defenses in the digestive system

Nucleotide sequences coding for *C*. *cumulans* SOD and GPX were identified from a partial RNA-Seq transcriptome of salivary gland and midgut cells (data not shown). Only one copy of each gene was identified in the transcriptome assembly. Their translated amino acid sequences exhibited remarkable similarity with other insect sequences when aligned (Figs S3 and S4, respectively), including conserved domains in the respective protein families. The SOD protein sequence showed 93% amino acid identity (blast score = 293 bits, e-value = 6e–98) to a SOD1 of *Coptotermes gestroi* and 92% identity (blast score = 290 bits, e-value = 7e–92) to a Cu/Zn SOD from the termite *Coptotermes formosanus*. GPX protein sequence showed 87% amino acid identity (blast score = 360 bits, e-value = 2e–124) to a GPX isoform X1 of *Zootermopsis nevadensis* and 74% identity (blast score = 273 bits, e-value = 5e–90) to a phospholipid hydroperoxide GPX from the coleopteran *Tribolium castaneum*. The relative abundance of mRNA and enzymatic activities of these antioxidant defenses were evaluated along the *C*. *cumulans* digestive system. Because it was the main site for DHE and CM-H_2_DCFDA oxidation, the midgut was used as the reference sample for relative abundance analyses.

**Figure 3:**
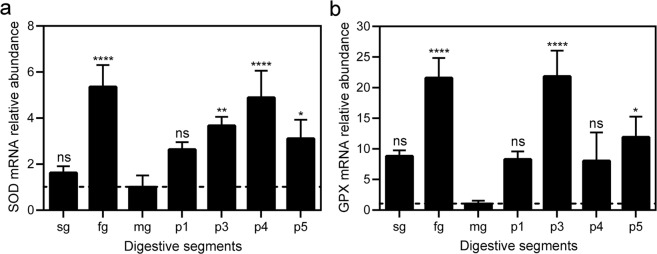
Relative abundance of antioxidant enzyme mRNAs along *C*. *cumulans* digestive system. (**a**) SOD mRNA levels; ns = no significance, ****p < 0.0001, **p = 0.0085 and *p = 0.0438. (**b**) GPX mRNA levels; ns = no significance, ****p < 0.0001 and *p = 0.0249. Salivary glands (sg), foregut (fg), midgut (mg) and hindgut compartments – p1, p3, p4 and p5. Analyses were performed using qRT-PCR and the 2^−ΔΔCT^ method, with HSP-70 as the housekeeping gene and the midgut as a reference sample. The data shown are mean ± S.E.M. from nine replicates with ANOVA and Dunnett’s multiple comparisons test.

The highest abundance of SOD mRNA was found in the foregut and hindgut p4 segment, where it was 4.3- and 3.9-fold higher than midgut, respectively. Lower abundances were detected in other hindgut compartments and salivary glands (Fig. 3a). Transcript levels of GPX were most abundant in hindgut p3 segment and foregut, each 20-fold greater than midgut. Lower levels were observed in other hindgut compartments and salivary glands (Fig. 3b).

Measurement of SOD and GPX enzymatic activities showed a similar pattern as that noted from mRNA levels. SOD activity was greatest in hindgut p5 compartment, lower in foregut and other hindgut segments, and smallest in the salivary glands and midgut (Table 2). The GPX activity was similar to that of SOD, being highest in hindgut p5 compartment and lowest in the salivary glands and midgut (Table 2).

**Table 2:** SOD and GPX activities along *C*. *cumulans* digestive system.

Segments	SOD (U/mg)	GPX (U/mg)
sg	0.34 ± 0.04	9.4 ± 0.7
fg	0.73 ± 0.08*	22.13 ± 1.93*
mg	0.35 ± 0.04	8.43 ± 0.8
p1	0.63 ± 0.07	21.03 ± 2.38
p3	0.74 ± 0.11*	21.41 ± 2.37
p4	0.59 ± 0.04	20.05 ± 1.08
p5	1.09 ± 0.16***	36.84 ± 8.16**

Values are mean ± S.E.M. from six samples of three different termite mounds (two replicates each and a pool of 40 termites per replicate). The values of enzymatic activities of each segment were compared to that of mg using Kruskal-Wallis and Dunn’s multiple comparisons test. U = 1 µmol reduced cytochrome *c* (SOD activity –fg *p = 0.0409, p3 *p = 0.0438 and ***p = 0.0006) or 1 µmol NADPH consumption (GPX activity – *p = 0.0228 and **p = 0.0027) per mg protein. Salivary glands (sg), foregut (fg), midgut (mg), hindgut compartments – p1, p3, p4 and p5, no significance (ns).

### Mitotic activity of midgut

**Figure 4:**
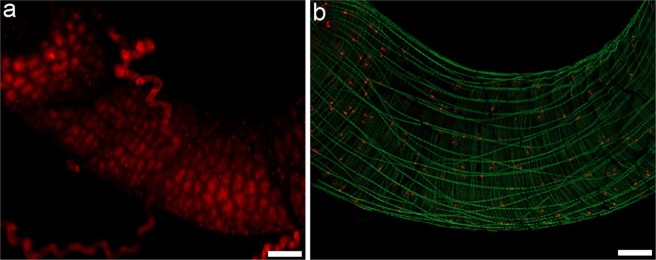
*C*. *cumulans* midgut as an oxidative and mitotically active environment. (**a**) Midgut labelled with DHE. Analysis under a confocal microscope employing the “z-stack” function and overlap of 350 µm. (**b**) Midgut mitotic cells revealed by anti-pHH3 antibody (red) and Alexa Fluor 488 phalloidin (green) highlighting muscle fibers. Analysis under a fluorescence stereomicroscope employing the “z-stack” function and overlap of 350 µm. Bars = 100 μm.

Among the *C*. *cumulans* gut compartments, the midgut was the main site for oxidant levels (Fig. 4a). However, it presented the smallest SOD and GPX mRNA levels (Fig. 3) and enzymatic activities (Table 2). Midgut epithelial proliferation is a well-known homeostatic response to a variety of tissue damages, including those caused by free radicals^40,41^. Therefore, regenerative activity was evaluated by measuring epithelial mitotic activity using fluorescence microscopy and a mitosis marker anti-pHH3 antibody. Higher numbers of mitotic cells were observed in the midgut (Fig. 4b – additional images are provided in Fig. S5) but was lacking in all other gut compartments (Fig. S6).

### Microbiota abundance in gut compartments

**Figure 5:**
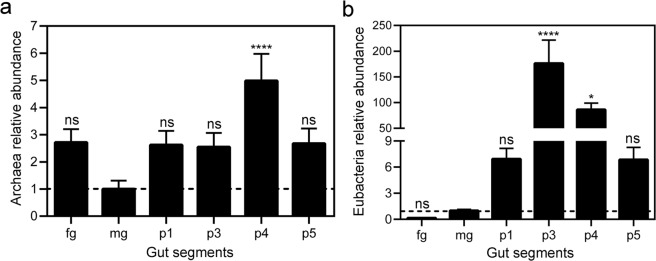
Relative abundance of Archaea and Eubacteria 16 S ribosomal DNA along *C*. *cumulans* alimentary canal. (**a**) Archaea abundance; ns = no significance and ****p < 0.0001. (**b**) Eubacteria abundance; ns = no significance, ****p < 0.0001 and *p = 0.0123. Foregut (fg), midgut (mg) and hindgut compartments – p1, p3, p4 and p5. Analyses were performed using qPCR and the 2^−ΔΔCT^ method, with termite HSP-70 as the housekeeping gene and the midgut as a reference sample. The data shown are mean ± S.E.M. from nine replicates with ANOVA and Dunnett’s multiple comparisons test.

qPCR analyses were used to determine the relative abundances of Archaea and Eubacteria throughout *C*. *cumulans* alimentary canal, employing the midgut as a reference sample. The hindgut p4 segment had the highest abundance of Archaea, 4-fold higher than the midgut. Despite having lower levels compared to p4 segment, the abundances of Archaea were higher in all the other gut compartments than in the midgut. Archaeal abundances were similar in the foregut, hindgut p1, p3, and p5 segments, which were approximately 1.5-fold higher than the abundance found in the midgut (Fig. 5a). For Eubacteria, much more variation was observed among the different tissues. Hindgut p3 segment showed the greatest abundance, 175-fold higher than the midgut, followed by the p4 compartment, 85-fold larger compared to midgut. The hindgut p1 and p5 segments had around 6-fold more Eubacteria than midgut, and the foregut presented the lowest abundance of these microorganisms (Fig. 5b).

## Discussion

While there are some studies describing the organization of the termite digestive system into different physicochemical microenvironments^37,42–49^, little is known about oxidant properties of their gut compartments. It has been proposed that non-enzymatic oxidative mechanisms might be involved in the digestive physiology of the termite guts^17,18^, either by creating an oxidative environment required for polyphenol-rich diet digestion or as a byproduct of the digestive process. Here, we observed the existence of variable levels of oxidants in some *C*. *cumulans* gut compartments. This led us to question how the gut cells protect themselves from potential oxidative damage and how oxidant molecules affect the distribution of microbiota along the alimentary canal. While trying to address these questions by comparing the physiology of the different gut compartments, we found unusual relationships between the levels of oxidants, antioxidant enzymatic defenses, gut epithelial cell renewal and microbiota abundance along the digestive system of the litter-feeding termite *C*. *cumulans*.

Considering that oxidants do not show substrate specificity regarding the molecules they can oxidize^14^, the different levels found along *C*. *cumulans* gut might be an indication of an oxidative process of food digestion. In this context, most of the oxidation occurs in the midgut, which had the highest level of oxidants and is spatially correlated with the region of the gut where hydrolytic digestion is known to occur in most Termitidae species^29,50^, including *C*. *cumulans* (data not shown). Alternatively, another important possibility for oxidant accumulation in the midgut is that they could be generated as byproducts of food processing. Furthermore, oxidants might also be generated by the epithelial cells as immune effectors, regulating the proliferation of microbiota in this gut compartment.

High levels of antioxidant enzymes (SOD and GPX) in the foregut and hindgut compartments probably are involved in the protection of the insect gut and its microbiota against potential oxidative injuries. Additionally, these compartments have cuticular layers protecting the luminal surface of the epithelial cells from abrasive and chemical damage^37^. On the contrary, high levels of oxidants in the midgut (an endodermic tissue devoid of cuticular layers) occurred together with low SOD and GPX transcript levels and enzymatic activities, where the cells seem to be more susceptible to oxidative damage.

Despite the presence of a peritrophic membrane in the midgut that is capable of protecting it from several types of injuries, including oxidative damage^25,26^, a high density of mitotic cells was identified only in the *C*. *cumulans* midgut, suggesting an elevated rate of epithelial regeneration in this region. Thereby, it seems likely that the building of an oxidative environment, which is proper for (or results from) the digestive process, rather than eliciting a physiological response from antioxidant defenses, is counteracted by increased rate of cell replacement. Similar responses were reported for other insect models where tissue damage induced a homeostatic increase in cell division^27,28^.

In the hindgut, p1 compartment showed no levels of oxidants and low densities of Archaea and Eubacteria, which might be associated with the extremely alkaline condition of this segment’s lumen^37^. Low oxidant levels in the hindgut p3 segment together with high SOD and GPX transcript levels and enzymatic activities probably account for the development of a propitious environment for the greatest abundance of Eubacteria. The eubacterial communities of termites’ p3 compartment have been shown to be important because of their fermentative metabolism generating acetate as the major energy substrate for termite metabolism^49^. The huge abundance of Eubacteria might also be related to the large volume of the p3 segment, which improves the fermentative processes by increasing the retention time of food particles at this region.

Therefore, in *C*. *cumulans* foregut, midgut and hindgut p3 compartment, there is an inverse relation between oxidant levels and microbiota abundance, a phenomenon also identified in the fruit fly^21^ and in the mosquito *Aedes aegypti*^23^. Indeed, it is widely accepted that oxidants are involved in antimicrobial responses by protecting insect guts from eubacterial infections^21^.

Alternatively, it is possible that some taxa could live in specific conditions (levels of O_2_, redox potentials, pH and others) along the several compartments of the gut. The p4 compartment, for example, showed high transcript levels and enzymatic activities of SOD and GPX, considerable levels of oxidants and high abundance of Eubacteria. Possibly, the Eubacterial taxa of the p4 segment may differ from those in other segments and have the ability to tolerate oxidants. The p4 compartment was also the main site of Archaea abundance. In termites, the archaeal microorganisms are extremophiles^32^ and play an important role in methane production, consuming the hydrogen generated by the fermentative processes of eubacterial communities^17,32,33^.

Interestingly, despite the p5 compartment having intermediate oxidant levels, low antioxidant mRNA levels and low microbiota abundance, it was the major site for SOD and GPX activities. As the last compartment of the alimentary canal, p5 epithelial cells must be overprotected from the reactivity of the digestion byproducts that are generated along all the other gut segments.

Despite the known role of oxidants in the degradation of lignocellulose and polyphenols, few studies have focused on the oxidative aspects of termite digestion. Together, our results provide new insights into litter-feeding termite digestive physiology emphasizing a unique management of oxidant levels and antioxidant defenses integrated with gut cell regeneration and microbiota abundance. In this fashion, the oxidative challenges imposed by polyphenol-rich diets seem to be well circumvented by *C*. *cumulans* gut, ensuring efficiency of the digestive process together with preservation of tissue homeostasis. However, new studies are needed to correlate oxidant levels with lignocellulose/polyphenol deconstruction and/or other physiological processes beyond digestion, such as immunity and stress response.

## Materials and Methods

### Termites

Nest fragments of three *C*. *cumulans* mounds were collected in Pinheiral grasslands, Rio de Janeiro, Brazil (GPS data: −22.5212761 and −43.9990613) and kept in polypropylene containers, in the dark at 23 ± 2 °C and 60 ± 10% relative humidity, with a mixture of litter and soil from the collection area as food. As social insects, workers are the main food decomposers of the termite mound. Hence, experiments were performed using only worker caste members of undetermined age and with completely full guts 10 days post-collection.

### Size and dry weight measurements of digestive segments

For size determination of each gut compartment, 10 worker guts were dissected and their segments (foregut, midgut, mixed segment, hindgut p1, p2, p3, p4 and p5 compartments) were measured under a stereomicroscope using a millimetre ruler. For dry weight measurements, salivary glands and gut compartments (with and without the food bolus) were isolated from 30 workers (for each replicate), dried at 50 °C for 48 h and weighed. Two replicates were tested for each collected termite mound, resulting in six replicates for dry weight experiments.

### Oxidant levels determination along the alimentary canal

To assess oxidant levels, 10 worker guts were dissected and incubated with a solution of oxidant-sensitive probes, 2 µM CM-H_2_DCFDA (5-(and-6)-chloromethyl-29,79-dichloride-dihydrofluorescein diacetate, acetyl ester) or 25 µM DHE (dihydroethidium) (Molecular Probes^™^, Thermo Fisher Scientific) in Schneider’s medium according to a previously published protocol^23^. After 20 min of incubation at room temperature in the dark, guts were washed in probe-free medium and transferred to a glass slide with a drop of Schneider’s medium. The slides were analysed under an Olympus Macroview MVX10 stereomicroscope equipped with an Olympus DP-72 colour CCD camera and a filter set for DHE (Ex/Em: 518/605 nm) or CM-H_2_DCFDA fluorescence (Ex/Em: ~492–495/517–527 nm). Alternatively, images were taken using a Zeiss LSM 510 META confocal microscope. Samples without fluorescent probes were analysed for autofluorescence control, using the same image acquisition conditions used in the experimental samples.

### Identification of gene sequences coding for antioxidant enzymes

A partial transcriptome (454 Roche platform) of the *C*. *cumulans* digestive system (unpublished data, previously sequenced and currently under analysis) was searched for SOD and GPX nucleotide sequences. The selected sequences were evaluated using BLASTX algorithm and employed for qRT-PCR experiments. Multiple sequence alignments were prepared using the Clustal Omega program (http://www.ebi.ac.uk/Tools/msa/clustalo/) and are presented as Box Shade Server (http://www.ch.embnet.org/software/BOX_form.html). Nucleotide sequences were deposited in European GenBank under the accession numbers: LS992199 (GPX), LS992200 (SOD) and LS992201 (HSP70).

### RNA extraction and qRT-PCR

To evaluate SOD and GPX transcript levels in the *C*. *cumulans* digestive system, salivary glands and gut compartments (foregut, midgut, mixed segment + hindgut p1, p3, p4 and p5 segments) were dissected from 30 workers (for each replicate) and immediately placed on ice. To minimize microbiota contamination, the food bolus was removed during dissection. Total RNA was extracted using Trizol^®^ reagent (Ambion^™^) according to the manufacturer’s standard protocol, precipitated with 1.4 M NaCl and re-purified using the RNeasy Mini kit (Qiagen) following manufacturer’s recommendations. RNA was subjected to RNase-free DNase I (Invitrogen^™^) treatment to remove any potential DNA contamination, and complementary DNA (cDNA) was synthesized using the High-Capacity cDNA Reverse Transcription Kit (Applied Biosystems^™^) following the manufacturer’s standard protocol. qPCR was carried out with Power SYBR^™^ Green PCR Master Mix (Applied Biosystems^™^) in a StepOnePlus^™^ Real-Time PCR System (Applied Biosystems^™^) under the following parameters: 95 °C for 10 min followed by 40 cycles of 95 °C for 15 sec and 60 °C for 1 min. The 2^−ΔΔCT^ method^51^ was adopted to calculate the relative abundance of the samples employing HSP-70 as a housekeeping gene. HSP-70 was selected after suitability tests between HSP-70, 18 S ribosomal protein and β-actin performed using BestKeeper^52^ and NormFinder^53^ software. The primer sequences used were: SOD 5′-CACACCATTGCTGTCAGCTT-3′ and 5′-TCCATGAATTTGGGGACAAT-3′, GPX 5′-TCCCAGAGTTCCTCCTTGCT-3′ and 5′-TGCCAAGTCCAAGAATGCCA-3′, and HSP-70 5′-CCGGCTGGTTGTCAGAGTAT-3′ and 5′-GCCATTTTGGCTGGAGATAA-3′. For relative abundance analyses, the midgut was used as the reference sample; all the digestive segments had their transcript levels compared to that of the midgut. Three replicates were examined for each collected termite mound; thus, nine replicates were employed in qRT-PCR experiments.

### Digestive extract preparation for enzymatic activity assays

For enzymatic activity assays, salivary glands and gut compartments (foregut, midgut, mixed segment + hindgut p1, p3, p4 and p5 segments) were dissected from 40 workers (for each replicate) and immediately frozen on dry ice. To reduce microbiota contamination, the food bolus was removed upon dissection. The samples were homogenized in 100 µl of 100 mM potassium phosphate buffer (KPB, pH 7.4) containing a general protease inhibitor cocktail (Sigma-Aldrich^®^) and centrifuged for 5 min at 20000 × g and 4 °C. The supernatants were collected, stored at −70 °C and used for the following enzymatic activity assays. Protein concentrations were estimated as previously described^54^, using bovine serum albumin as a standard. Two replicates were tested for each of the three termite mounds, resulting in six replicates for enzymatic assays.

### Superoxide dismutase activity

The production of superoxide by a xanthine-xanthine oxidase system and concomitant cytochrome *c* reduction was used to quantify SOD activity^55^ in the *C*. *cumulans* digestive system. Reactions were carried out in 1 ml cuvettes in the presence of 50 mM KPB (pH 7.4), 0.1 mM EDTA, 500 µM xanthine, 0.005 U xanthine oxidase, 20 µM oxidized cytochrome *c* and initiated by the addition of 20 µl digestive extracts. The rate of cytochrome *c* reduction was monitored for 6 min at 550 nm in a Shimadzu UV-2550 spectrophotometer. Negative control (blank) was run in the absence of digestive extracts.

### Glutathione peroxidase activity

GPX activity was assayed by measuring the amount of oxidized NADPH (NADP+), coupling reduced glutathione (GSH) production to the reaction catalysed by glutathione reductase (GR)^56^. GSH was reduced from GSSG (oxidation of GSH) generated by GPX. The reactions were performed in 1 ml cuvettes containing 100 mM KPB (pH 7.4), 200 mM NADPH, 1 mM GSH, 1 U GR, 20 µl *C*. *cumulans* digestive extracts, and initiated by the addition of 17 µM tert-butyl hydroperoxide or 100 μM H_2_O_2_. NADPH consumption was followed during 6 min at 340 nm in a Shimadzu UV-2550 spectrophotometer. Negative control (blank) was run without digestive extracts.

### Immunohistochemistry of mitotic cells

The presence of mitotic cells was determined as previously described^37^, using a mouse anti-phospho-Histone H3 (anti-pHH3; Merck Millipore) primary antibody and goat anti-mouse Alexa Fluor 546 conjugate (Thermo Fisher Scientific^™^) as the secondary antibody and co-staining with Alexa Fluor 488 phalloidin (Thermo Fisher Scientific^™^). Images were acquired with a Zeiss AxioZoom.V16 stereo zoom microscope. Alternatively, images were taken with a Leica TCS-SPE laser scanning confocal microscope.

### Determination of microbiota abundance in gut compartments

To determine the microbiota abundance along the *C*. *cumulans* alimentary canal, gut compartments (foregut, midgut, mixed segment + hindgut p1, p3, p4 and p5 segments) were dissected from 10 workers (of each replicate) and immediately placed on ice. DNA was extracted using PowerSoil^®^ DNA Isolation Kit (MoBio) under manufacturer’s standard protocol and used for archaeal and eubacterial 16 S ribosomal DNA relative quantification by qPCR with Power SYBR^™^ Green PCR Master Mix (Applied Biosystems^™^), as described above. Primer pairs were designed according to previously published data^57^, based on Archaea and Eubacteria predicted phyla along the *C*. *cumulans* gut, as discussed previously^38,39^. These were: Archaea (A346-361) 5′-GGGGYGCAGCAGGCG-3′ and (A519-539) 5′-GYGGTDTTACCGCGGCGGCTG-3′ and Eubacteria (E969-984) 5′-ACGCGARGAACCTTAC-3′ and (E1063-1081) 5′-CTCACGRCACGAGCTGACG-3′. The midgut was used as the reference sample to estimate relative abundance. Three replicates were examined for each collected *C*. *cumulans* mound; hence, nine replicates were used in this experiment.

### Statistical analysis

The statistical analyses were performed using one-way analysis of variance (ANOVA) and Dunnett’s multiple comparisons test, with a 95% confidence interval, after a Gaussian distribution test using normality tests (GraphPad Prism 6). For non-parametric data, the statistical analyses were performed using Kruskal-Wallis and Dunn’s multiple comparisons test.

## Supplementary information

Supplementary information

## References

[1] Himmel ME (2007). Biomass recalcitrance: engineering plants and enzymes for biofuels production. Science..

[2] Rubin EM (2008). Genomics of cellulosic biofuels. Nature..

[3] Scharf ME, Boucias DG (2010). Potential of termite based biomass pre-treatment strategies for use in bioethanol production. Insect Sci..

[4] Scharf ME, Tartar A (2008). Termite digestomes as sources for novel lignocellulases. Biofuels Bioprod. Biorefin..

[5] Ohkuma M (2003). Termite symbiotic systems: efficient bio recycling of lignocellulose. Appl. Microbiol. Biotechnol..

[6] Cragg SM (2015). Lignocellulose degradation mechanisms across the Tree of Life. Curr. Opin. Chem. Biol..

[7] Franco Cairo JPL (2016). Expanding the knowledge on lignocellulolytic and redox enzymes of worker and soldier castes from the lower termite. Coptotermes gestroi. Front. Microbiol..

[8] Martinez D (2009). Genome, transcriptome, and secretome analysis of wood decay fungus *Postia placenta* supports unique mechanisms of lignocellulose conversion. Proc. Natl. Acad. Sci. USA.

[9] Tartar A (2009). Parallel metatranscriptome analyses of host and symbiont gene expression in the gut of the termite *Reticulitermes flavipes*. Biotechnol. Biofuels..

[10] Warnecke F (2007). Metagenomic and functional analysis of hindgut microbiota of a wood-feeding higher termite. Nature..

[11] Coy MR (2010). Phenol-oxidizing laccases from the termite gut. Insect Biochem. Mol. Biol..

[12] Bak JS (2015). Lignocellulose depolymerization occurs via an environmentally adapted metabolic cascades in the wood-rotting basidiomycete *Phanerochaete chrysosporium*. Microbiologyopen..

[13] Baldrian P, Valášková V (2008). Degradation of cellulose by basidiomycetous fungi. FEMS Microbiol. Rev..

[14] Halliwell, B. & Gutteridge, J. M. C. *Free radicals in biology and medicine*. Oxford University Press, New York, USA (2007).

[15] Barbehenn RV, Bumgarner SL, Roosen EF, Martin MM (2001). Antioxidant defenses in caterpillars: role of the ascorbate-recycling system in the midgut lumen. J. Insect Physiol..

[16] Shah V, Nerud F (2002). Lignin degrading system of white-rot fungi and its exploitation for dye decolorization. Can. J. Microbiol..

[17] Brune A (2014). Symbiotic digestion of lignocellulose in termite guts. Nat. Rev. Microbiol..

[18] Vu AT, Nguyen NC, Leadbetter JR (2004). Iron reduction in the metal-rich guts of wood-feeding termites. Geobiology..

[19] Krishnan N, Kodrík D (2006). Antioxidant enzymes in *Spodoptera littorali*s(Boisduval): Are they enhanced to protect gut tissues during oxidative stress?. J. Insect Physiol..

[20] Summers CB, Felton GW (1994). Prooxidant effects of phenolic acids on the generalist herbivore *Helicoverpa zea* (Lepidoptera: Noctuidae): Potential mode of action for phenolic compounds in plant anti-herbivore chemistry. Insect Biochem. Mol. Biol..

[21] Ha EM, Oh CT, Bae YS, Lee WJ (2005). A direct role for dual oxidase in *Drosophila* gut immunity. Science..

[22] Kumar S, Molina-Cruz A, Gupta L, Rodrigues J, Barillas-Mury C (2010). A peroxidase/dual oxidase system modulates midgut epithelial immunity in *Anopheles gambiae*. Science..

[23] Oliveira JHM (2011). Blood meal-derived heme decreases ROS levels in the midgut of *Aedes aegypti* and allows proliferation of intestinal microbiota. PLoS Pathog..

[24] Winterbourn CC (2008). Reconciling the chemistry and biology of reactive oxygen species. Nat. Chem. Biol..

[25] Sandoval-Mojica AF, Scharf ME (2016). Gut genes associated with the peritrophic matrix in *Reticulitermes flavipes* (Blattodea: Rhinotermitidae): Identification and characterization. Arch. Insect Biochem. Physiol..

[26] Sandoval-Mojica AF, Scharf ME (2016). Silencing gut genes associated with the peritrophic matrix of *Reticulitermes flavipes* (Blattodea: Rhinotermitidae) increases susceptibility to termiticides. Insect Mol. Biol..

[27] Jiang H, Tian A, Jiang J (2016). Intestinal stem cell response to injury: lessons from *Drosophila*. Cell. Mol. Life Sci..

[28] Scully ED, Hoover K, Carlson JE, Tien M, Geib SM (2013). Midgut transcriptome profiling of *Anoplophora glabripennis*, a lignocellulose degrading cerambycid beetle. BMC Genom..

[29] Watanabe H, Tokuda G (2010). Cellulolytic systems in insects. Ann. Rev. Entomol..

[30] Bignell, D. E. Morphology, physiology, biochemistry and functional design of the termite gut: an evolutionary wonderland. In *Biology of termites*: *a modern synthesis* (eds Bignell, D. E., Roisin, Y. & Lo, N.) 375–412 (Springer, Dordrecht, 2011).

[31] Breznak, J. A. Ecology of prokaryotic microbes in the guts of wood- and litter-feeding termites. In *Termites*: *evolution*, *sociality*, *symbiosis*, *ecology* (eds Abe, T., Bignell, D. E. & Higashi, M.) 209–231 (Springer, Dordrecht, 2000).

[32] Brune, A. Methanogenesis in the digestive tracts of insects. In *Handbook of hydrocarbon and lipid microbiology* (ed. Timmis, K. N.) 707–728 (Springer, Heidelberg, 2010a).

[33] Brune, A. Methanogens in the digestive tract of termites. In *(Endo) symbiotic methanogenic Archaea* (ed. Hackstein, J. H. P.) 81–100 (Springer, Heidelberg, 2010b).

[34] Brune, A. & Ohkuma, M. Role of the termite gut microbiota in symbiotic digestion. In *Biology of termites: a modern synthesis* (eds Bignell, D. E., Roisin, Y. & Lo, N.) 439–475 (Springer, Dordrecht, 2011).

[35] Franco Cairo JPL (2011). Functional characterization and target discovery of glycoside hydrolases from lower termite. Coptotermes gestroi. Biotechnol. Biofuels..

[36] Donovan SE, Eggleton P, Bignell DE (2001). Gut content analysis and a new feeding group classification of termites. Ecol. Entomol..

[37] Sousa G (2017). Morphophysiological study of digestive system litter-feeding termite *Cornitermes cumulans* (Kollar, 1832). Cell Tissue Res..

[38] Costa PS, Oliveira PL, Chartone-Souza E, Nascimento AMA (2013). Phylogenetic diversity of prokaryotes associated with the mandibulate nasute termite *Cornitermes cumulans* and its mound. Biol. Fertil. Soils..

[39] Grieco MAB (2013). Microbial community diversity in the gut of the South American termite *Cornitermes cumulans* (Isoptera: Termitidae). Microb. Ecol..

[40] Barbehenn RV, Stannard J (2004). Antioxidant defense of the midgut epithelium by the peritrophic envelope in caterpillars. J. Insect Physiol..

[41] Nászai M, Carroll LR, Cordero JB (2015). Intestinal stem cell proliferation and epithelial homeostasis in the adult *Drosophila* midgut. Insect Biochem. Mol. Biol..

[42] Bignell DE, Eggleton P (1995). On the elevated intestinal pH of higher termites (Isoptera: Termitidae). Insect Soc..

[43] Brune A, Emerson D, Breznak JA (1995). The termite gut microflora as an oxygen sink: microelectrode determination of oxygen and pH gradients in guts of lower and higher termites. Appl. Environ. Microbiol..

[44] Brune A, Kühl M (1996). pH profiles of the extremely alkaline hindguts of soil-feeding termites (Isoptera: Termitidae) determined with microelectrodes. J. Insect Physiol..

[45] Schmitt-Wagner D, Brune A (1999). Hydrogen profiles and localization of methanogenic activities in the highly compartmentalized hindgut of soil-feeding higher termites (*Cubitermes* spp.). Appl. Environ. Microbiol..

[46] Brune A, Friedrich MW (2000). Microecology of the termite gut: structure and function on a microscale. Curr. Opin. Microbiol..

[47] Friedrich MW, Schmitt-Wagner D, Lueders T, Brune A (2001). Axial differences in community structure of Crenarchaeota and Euryarchaeota in the highly compartmentalized gut of the soil-feeding termite *Cubitermes orthognathus*. Appl. Environ. Microbiol..

[48] Schmitt-Wagner D, Friedrich MW, Wagner B, Brune A (2003). Axial dynamics, stability, and interspecies similarity of bacterial community structure in the highly compartmentalized gut of soil-feeding termites (*Cubitermes* spp.). Appl. Environ. Microbiol..

[49] Köhler T, Dietrich C, Scheffrahn RH, Brune A (2012). High-resolution analysis of gut environment and bacterial microbiota reveals functional compartmentation of the gut in wood-feeding higher termites (*Nasutitermes* spp.). Appl. Environ. Microbiol..

[50] Ni J, Tokuda G (2013). Lignocellulose-degrading enzymes from termites and their symbiotic microbiota. Biotechnol. Adv..

[51] Livak KJ, Schmittgen TD (2011). Analysis of relative gene expression data using real-time quantitative PCR and the 2(-Delta C(T)) method. Methods..

[52] Pfaffl MW, Tichopad A, Prgomet C, Neuvians TP (2004). Determination of stable housekeeping genes, differentially regulated target genes and sample integrity: BestKeeper – excel-based tool using pair-wise correlations. Biotechnol. Lett..

[53] Andersen CL, Jensen JL, Ørntoft TF (2004). Normalization of real-time quantitative reverse transcription-PCR data: a model-based variance estimation approach to identify genes suited for normalization, applied to bladder and colon cancer data sets. Cancer Res..

[54] Lowry OH, Rosebrough NJ, Farr AL, Randall RJ (1951). Protein measurement with the folin phenol reagent. J. Biol. Chem..

[55] Flohé L, Ötting F (1984). Superoxide dismutase assay. Methods Enzymol..

[56] Paglia DE, Valentine WN (1967). Studies on the quantitative and qualitative characterization of erythrocyte glutathione peroxidase. J. Lab. Clin. Med..

[57] Wang Y, Qian PY (2009). Conservative fragments in bacterial 16S rRNA genes and primer design for 16S ribosomal DNA amplicons in metagenomic studies. PLoS One..

