# Supplementary material for: The relationship between oxidant levels and gut physiology in a litter-feeding termite
Source: Sci Rep. 2019 Jan 24;9:670. doi: 10.1038/s41598-018-37043-2 (PMC6345907; doi:10.1038/s41598-018-37043-2)
Supplement: Supplementary file 1 — Supplementary information [file 41598_2018_37043_MOESM1_ESM.pdf]

Supplementary information

Manuscript: The relationship between oxidant levels and gut physiology in a litter-feeding termite

Authored by: Gessica Sousa, Ana Caroline P. Gandara, Pedro L. Oliveira, Fabio M. Gomes, Ana Cristina Bahia, Ednildo A. Machado

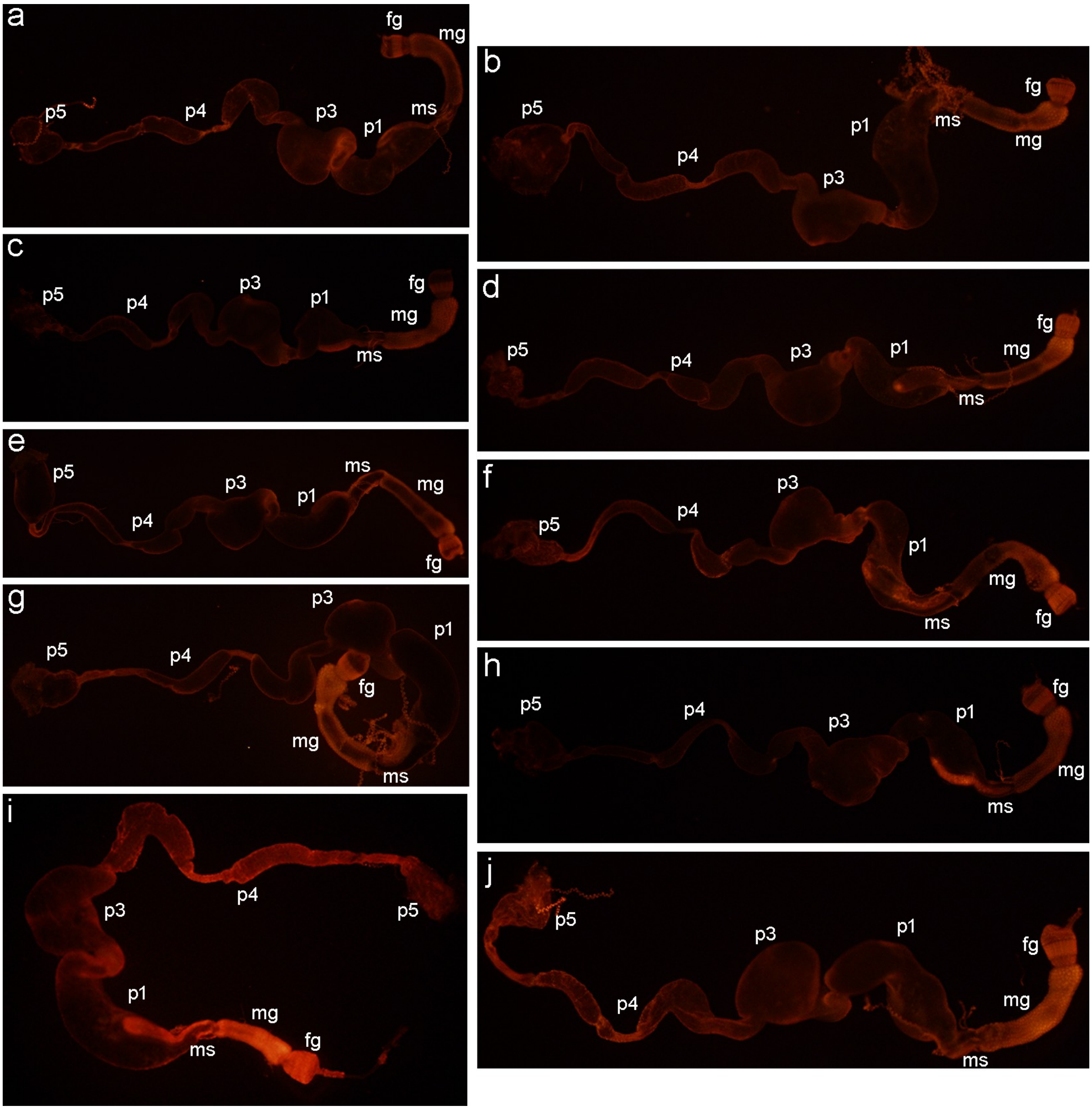

Fig. S1. Additional images to straight the profile of oxidant levels along *C. cumulans* alimentary canal. The guts were incubated with DHE and analyzed under a fluorescence stereomicroscope. Foregut (fg), midgut (mg), mixed segment (ms) and hindgut compartments – p1, p3, p4 and p5.

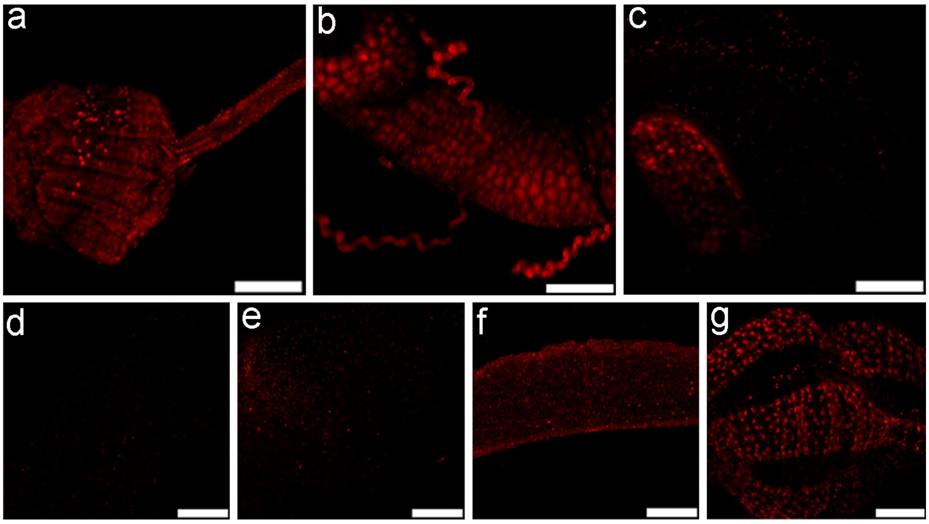

Fig. S2. Further details of oxidant levels along *C. cumulans* alimentary canal. Guts labelled with DHE and analyzed under a confocal microscope employing the "z-stack" function and overlap of 350 µm. **a** Foregut, **b** midgut, **c** mixed segment and hindgut **d** p1, **e** p3, **f** p4 and **g** p5 compartments. Bars = 100 µm.

```
ZnSOD     1 ---------------------------------------------MPVKAVCVLNGE
CcSOD     1 RRVAITWGGTRSCWSSLEVVQAGLVV*TFG*FTK*SLFQGTERKRPLIMPLKAVCVLKGE
CgSOD     1 ---------------------------------------------MPIKAVCVLNGE
CfSOD     1 ---------------------------------------------MPIKAVCVLNGE

ZnSOD    13 VVKGTLFFDQVNPDSPVRVTGEVTGLSQGLHGFHIHEFGDNTNGCISAGAHFNPHQKDHG
CcSOD    58 VVKGTVFFDQDDPESEVKVTGEVTGLSKGLHGFHIHEFGDNTNGCTSAGAHFNPYQKDHG
CgSOD    13 VVKGTLFFDQENPDSAVKVTGEVTGLSKGLHGFHIHEFGDNTNGCTSAGAHFNPYQKDHG
CfSOD    13 VVKGTLFFEQENPDSAVKVTGEVTGLSKGLHGFHIHEFGDNTNGCTSAGAHFNPYQKDHG

ZnSOD    73 SPTDEIRHVGDLGNIEAGSNGVAKVDITDKLISLSGAQNIIGRTLVVHADPDDLGKGGHE
CcSOD   118 GPDDQVRHVGDLGNVEADSNGVAKVDIADKLISLTGAHNIIGRTVVVHADPDDLGKGGHE
CgSOD    73 GPDDEVRHVGDLGNIEADSSGVAKVDIADKLISLTGAHNIIGRTLVVHADPDDLGKGGHE
CfSOD    73 GPDDEVRHVGDLGNIEADSSGVAKVDIADKVISLTGAHNIIGRTLVVHADPDDLGKGGHE

ZnSOD   133 LSKTTGNAGARSACGVVGIAKI--------------------------------------
CcSOD   178 LSKTTGNAGARSACGVVGIAKI*CMLHVKVFCLVVC*QRPGLACSWKFK*QCRLPSIK*V
CgSOD   133 LSKTTGNAGARSACGVVGIAKI--------------------------------------
CfSOD   133 LSKTTGNAGARSACGVVGIAKI--------------------------------------

ZnSOD       ------------------------------------
CcSOD   234 DSISAS*GTVS*CMI*IFIPYLCNKESHII*EKKKX
CgSOD       ------------------------------------
CfSOD       ------------------------------------
```

Fig. S3. Multiple amino acid sequence alignment of *C. cumulans* SOD with sequences from other insects. The protein was denominated Cc (*C. cumulans*) SOD and compared to *Zootermopsis nevadensis* (ZnSOD – KDR12362), *Coptotermes gestroi* (CgSOD – ALM23457) and *Coptotermes formosanus* (CfSOD – AGM32998). Identic amino acids (black), amino acids from the same classification group according to the side chain (grey) and codon coding for a termination amino acid (*). The highest score blast X matches to *C. cumulans* SOD was 8e-98 for SOD1 (*C. gestroi*). Sequence ID: ALM23457.1.

```
RpGPX     1 --------------------------------------------------MRYYLLIVSL
TdGPX     1 ------------------------------------------------------------
CcGPX     1 RVPITAGGRREV*GSVGRVHIDW*GCCRQVFVSI*TAIAEERHNFTSVKEFAYKVKSEGL
ZnGPX     1 ------------------------------------------------------MLVPGL

RpGPX    11 ILLATSIILH-YQLTGFYSTEHTMAESEKDASSVYDFTVKDIAGNDISLEKYRGHVLIIV
TdGPX     1 ---------------------RTMATSEKDATSVYDFTVKDISGNDVSLEKYRGHVLIIV
CcGPX    58 QLFGAAFCALRLLSTRAAPVMAATPEAWKNASSIYEFTVKDINGQDVSLEKYRGHVAVIV
ZnGPX     7 QLFGTALCALRLLSTRTSPVMASTPEDWKNASSIYDFTVKDINGQDVSLEKYKGHAAIIV

RpGPX    70 NVASQCGLTSTNYKELVELDEKYRETKGLRILAFPCNQFGSQEPGSREDIVCFMRQKNAS
TdGPX    40 NVASQCGLTSTNYKELVELDEKYRDTKGLRILAFPCNQFGGQEPGSREDIVCFMKQKNAT
CcGPX   118 NVASKCGFTPTNYKELAELHDQYAESKGLKILAFPCNQFNGQEPGTPEEIVCFAKSKNAK
ZnGPX    67 NVASKCGLTATNYKELAELHDNYADSKGLRILAFPCNQFNSQEPGNSEEIVCFAKSKNAK

RpGPX   130 FEMFDKIEVNGSNAHPLWKYLKSKQGGTLVDNIKWNFTKFIIDKNGQPVERHGPMTNPSK
TdGPX   100 FEMFDKIEVNGNNAHPLWKYLKSKQGGTLVDNIKWNFTKFIIDKNGQPVERHGPMTNPSK
CcGPX   178 FDMFEKIDVNGKNAHPLWKYLEYKQGGTLGDFIKWNFTKFIIDKNGQPVERHGPNVDPSK
ZnGPX   127 FDLFEKIDVNGNNSHPLWKYLKYKQGGTLGDFIKWNFTKFIIDKNGQPVERHGPNVDPAK

RpGPX   190 LLSSLEKYW--------
TdGPX   160 LVSCLEKYW--------
CcGPX   238 LVSSFEKYW*CP*RYTX
ZnGPX   187 LVSNLEKYW--------
```

Fig. S4. Multiple amino acid sequence alignment of *C. cumulans* GPX with sequences from other insects. The protein was denominated Cc (*C. cumulans*) GPX and compared to *Rhodnius prolixus* (RpGPX – JAA75589), *Triatoma dimidiata* (TdGPX – JAP03111) and *Zootermopsis nevadensis* (ZnGPX – KDR22003). Identic amino acids (black), amino acids from the same classification group according to the side chain (grey) and codon coding for a termination amino acid (*). The highest score blast X matches to *C. cumulans* GPX was 4e-124 for phospholipid hydroperoxide GPX isoform X1 (Z. *nevadensis*). Sequence ID: XP_021914630.1.

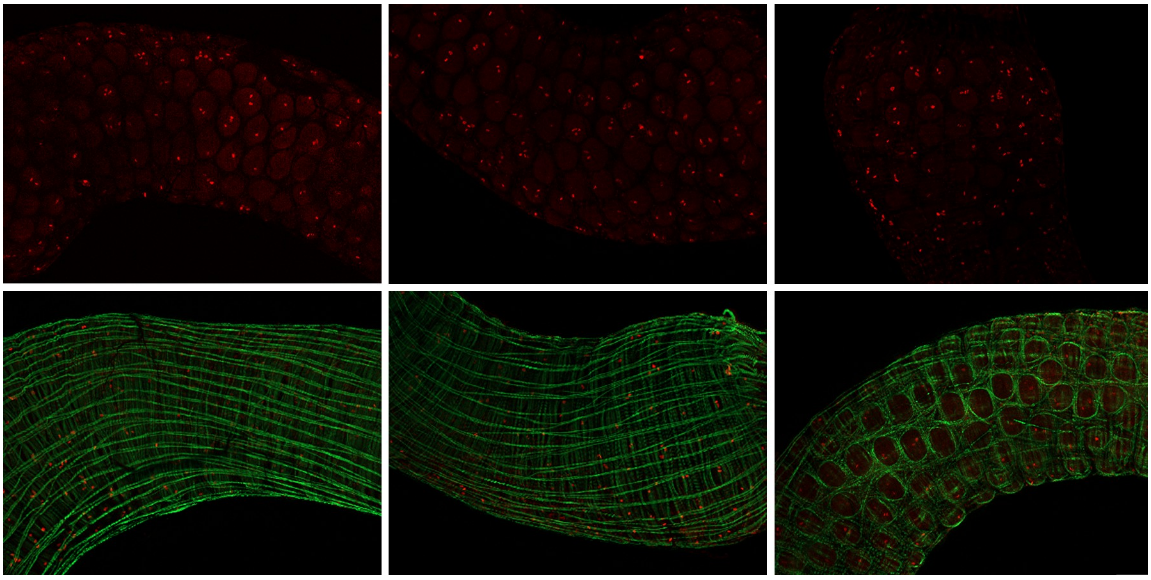

Fig. S5. Additional images to straight the profile of midgut cell renewal. The mitotic cells were revealed by anti-pHH3 antibody (red). The co-marking of Alexa Fluor 488 phalloidin (green) highlights muscle fibers. Analysis under a fluorescence stereomicroscope employing the “z-stack” function and overlap of 350 μm.

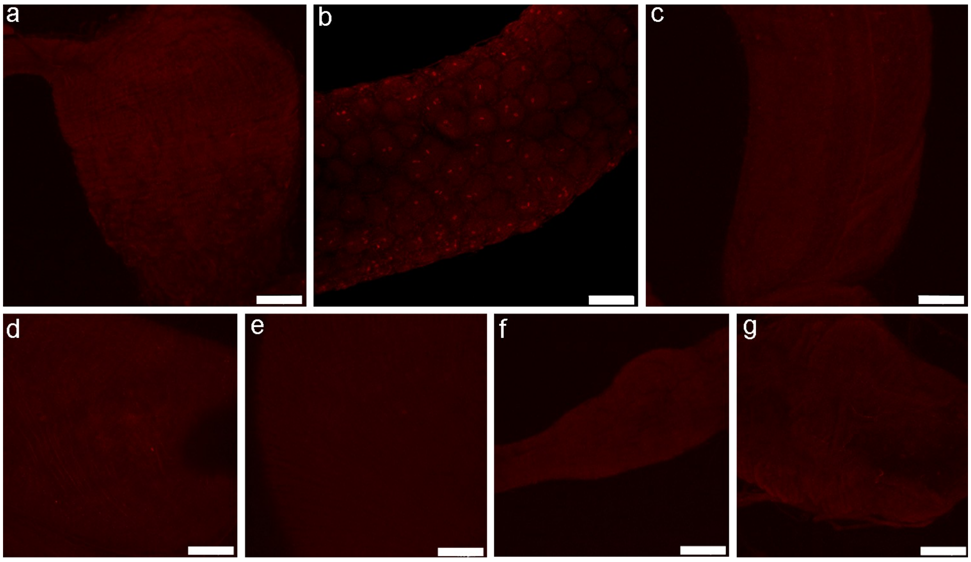

Fig. S6. Further details of mitotic profile of *C. cumulans* alimentary canal. Mitotic cells revealed by anti-pHH3 antibody (red) and analyzed under a confocal microscope employing the “z-stack” function and overlap of 350 µm. **a** Foregut, **b** midgut, **c** mixed segment and hindgut **d** p1, **e** p3, **f** p4 and **g** p5 compartments. Bars = 100 µm.
